# Integrating 3D Model Representation for an Accurate Non-Invasive Assessment of Pressure Injuries with Deep Learning

**DOI:** 10.3390/s20102933

**Published:** 2020-05-21

**Authors:** Sofia Zahia, Begonya Garcia-Zapirain, Adel Elmaghraby

**Affiliations:** 1 eVIDA Research Group, University of Deusto, 48007 Bilbao, Spain; mbgarciazapi@deusto.es; 2 Computer Science and Engineering Department, University of Louisville, Louisville, KY 40292, USA; adel@louisville.edu

**Keywords:** computer-assisted intervention, pressure injury, biomedical sensing, deep learning and diagnosis

## Abstract

Pressure injuries represent a major concern in many nations. These wounds result from prolonged pressure on the skin, which mainly occur among elderly and disabled patients. If retrieving quantitative information using invasive methods is the most used method, it causes significant pain and discomfort to the patients and may also increase the risk of infections. Hence, developing non-intrusive methods for the assessment of pressure injuries would represent a highly useful tool for caregivers and a relief for patients. Traditional methods rely on findings retrieved solely from 2D images. Thus, bypassing the 3D information deriving from the deep and irregular shape of this type of wounds leads to biased measurements. In this paper, we propose an end-to-end system which uses a single 2D image and a 3D mesh of the pressure injury, acquired using the Structure Sensor, and outputs all the necessary findings such as: external segmentation of the wound as well as its real-world measurements (depth, area, volume, major axis and minor axis). More specifically, a first block composed of a Mask RCNN model uses the 2D image to output the segmentation of the external boundaries of the wound. Then, a second block matches the 2D and 3D views to segment the wound in the 3D mesh using the segmentation output and generates the aforementioned real-world measurements. Experimental results showed that the proposed framework can not only output refined segmentation with 87% precision, but also retrieves reliable measurements, which can be used for medical assessment and healing evaluation of pressure injuries.

## 1. Introduction

**Figure 1:**
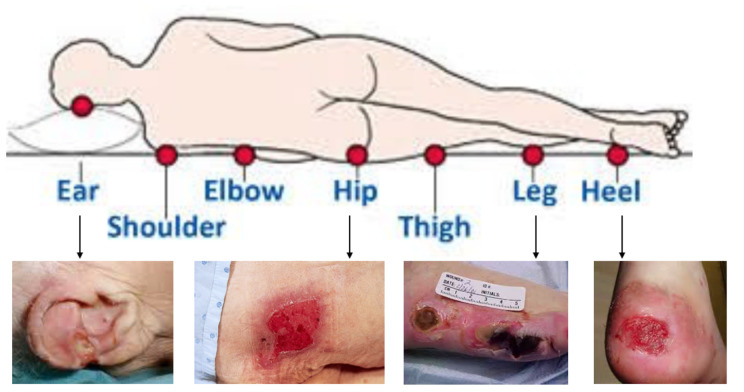
Body parts affected by pressure injuries [3].

Pressure injuries represent one of the greatest challenges in the healthcare sector, as they are considered one of the major causes of death, and a financial burden on healthcare systems [1,2]. They are chronic wounds resulting from damage caused by pressure over time causing an ischemia of underlying skin structures, especially on bony areas [3], as shown in Figure 1. Pressure injury stages varies from stage 1 to stage 4 depending on the deepest parts of the ulcer and the type of tissue affected. Higher stage ulcer represents deeper tissue damage and more serious injury. While previous sources estimated in 2011 the cost of the treatment of these wounds to be $9.1–11.6 billion per year in the US [4], recent claims by Medicare beneficiaries showed that chronic pressure injury care increased to about $22 billion [5], and the mean cost of treatment per patient varied between £1214 for Stage 1 pressure injuries, and up to £14,108 for stage Stage 4 in the UK [6].

The clinical practice to measure the wounds has not improved up to now. Previously, waterfill and cotton swab methods were largely used to measure the volume and depth of the wound. The depth is normally measured using the cotton swab from the deepest point, whereas the volume is measured from the volume of liquid inserted in the wound. These intrusive measurement methods not only cause pain and discomfort for the patients, but may also lead to serious infections.

For this very reason, non-intrusive methods are highly needed. The common method used to measure wounds area is using a ruler, which has poor inter-rater and intra-rater concordance. Given the irregular shape of these wounds, as well as their deep structure, especially those encountered in the sacrum, measuring the area using the longest and shortest length leads to variable inaccuracies. Thanks to technological progress, 3D object scanning has been made possible with the emergence of 3D scanning devices.

These tools have been widely used in several domains of application such as products and apparel designing as well as healthcare applications [7,8,9,10]. In our study, we used the Occipital Inc. (San Francisco, CA, USA) Structure Sensor (ST01) [11], a 3D scanning device to acquire the 3D meshes of the pressure injuries.

**Figure 2:**
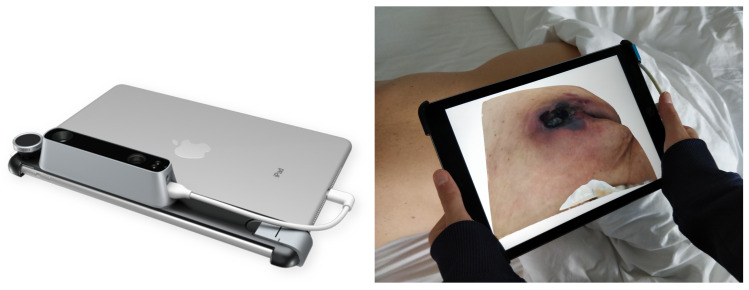
(**Left**) The Structure Sensor mounted on an iPad. (**Right**) Scanning of a pressure injury using the Structure Sensor.

The Structure Sensor is the first 3D scanner used on mobile devices. While it is performing the best with iOS devices, it can still be used with other devices and platforms, including Android devices, Windows, macOS and laptop/desktop computers. In our proposed research, we mounted it on an iPad Air 2, using its customized bracket. This Structured Light System (SLS) contains a laser-emitting diode, an infrared radiation range projector and an infrared sensor. Then, using a safe infra-red light, the sensor scans the objects and the iPad’s RGB camera sends data to a System On a Chip (SOC) for processing. The 3D models of the pressure injuries were captured using the “Scanner” app, by moving the device around the area, as shown in Figure 2.

The sensor alone delivers a point dataset, of a 640 × 480 pixels resolution, where each pixel contains the distance from the sensor to the target. The role of the infrared (IR) sensor is to record the reflectance intensity of the infrared light pattern projected by the IR projector onto the target, then the point cloud is triangulated by the SOC to form the 3D mesh [12].

The accuracy of the Structure Sensor has been studied and revealed the reliability of its usage for object 3D reconstruction. On one hand, Kalantari, M. et al. [12] present the accuracy of measurements from the Structure Sensor data, especially point clouds in the context of volunteered 3D indoor information, compared to the ground-truth. They achieved an error of 2.1 cm for the smallest room captured, which was from a distance of 2m at most. The authors show that the Structure Sensor is accurate for smaller rooms, and when the acquisition is made in a circular way. During the acquisition of the pressure injuries’ 3D meshes, we captured the wounds at a distance of 30 cm, by moving the sensor also around the area. This shows that the 3D reconstruction using the Structure Sensor is highly reliable. On the other hand, in [13], the authors showed that the 3D scanner gives accurate volumetric measurements in comparison to standard volumetric measurements obtained by the waterfill technique. In addition, the 3D scanner was found to be more reliable and valid compared to other three techniques: the ruler method, acetate grid tracing and 2D planimetric measurements.

## 2. Related Work

During the last decade, the information quality has highly increased as well as the storage infrastructure. Capturing high quality images has become feasible all over the world. Since healthcare professionals need to quantify the visual characteristics of the wounds, computer vision techniques combined with machine learning are the key solution.

Traditionally, wound image segmentation was performed using image processing techniques [3]. Several image processing approaches have been proposed in the literature, such as Region-based segmentation [14,15,16,17], and edge-based segmentation [14,18,19]. In addition, traditional machine learning methods were used to perform wound segmentation. Both unsupervised methods such as clustering [20,21], and supervised methods such as Support Vector Machines (SVM) [22,23] and Bayesian classifiers [24] were used to segment the wound after extracting the features such as mean-color information, color histogram statistics and Scale Invariant Feature Transform (SIFT) features [25]. The issue with traditional approaches is the necessity to choose the features which are important in the given images. Hence, the computer vision engineers need to judge with a trial and error process to decide the features which best describe the objects to detect. Deep learning has introduced the concept of end-to-end learning. When the machine is given a set of images, which have been annotated by experts, Neural networks discover the underlying patterns during the training and automatically find out the most descriptive features with respect to the objects to be detected. In fact, in the last decades, deep learning has become the dominant method for many machine vision-based applications such as object detection, object localization and semantic segmentation [26], especially Convolutional Neural Networks (CNNs), which have highly outperformed the traditional machine learning algorithms [27]. These methods have achieved state-of-the-art performance in a variety of medical image processing tasks, including medical image segmentation. However, to the best of our knowledge, there has been little research conducted on skin wound imaging using deep learning techniques [3]. Table 1 gives a summary of the contributions in the field of pressure injuries segmentation and measurement. Some of the related works were able to measure the segmentation area of the wound using real-world units [28,29,30]. However, bypassing the 3D information of the wounds results in biased values. On the other hand, our proposed method uses the 3D features of the pressure injuries and delivers reliable measurements, which are needed for an efficient assessment of these wounds by medical practitioners.

In this paper, we propose an end-to-end system which automatically segments the pressure injury and extracts all quantitative information by matching the 2D image with its 3D mesh. Our system first uses a Mask-RCNN architecture to tackle the segmentation task. Then using the rasterization of the 3D mesh, both the photograph of the wound and the top view are matched, and the segmentation result is used to segment the 3D mesh. Then a measurement pipeline is used to retrieve the depth, volume, areas, major and minor axes.

## 3. Materials and Methods

### 3.1. Global Framework

**Figure 3:**
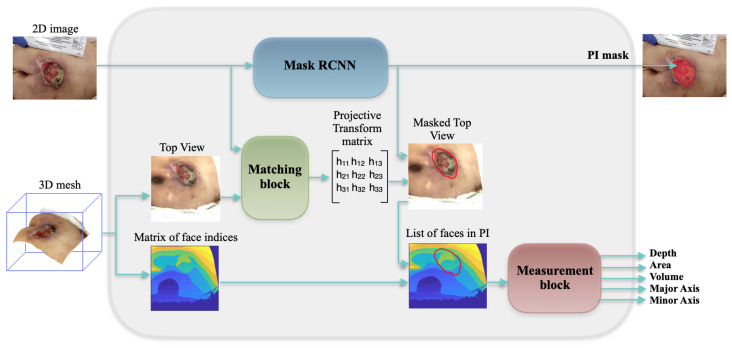
Schematic view of our proposed framework.

**Figure 4:**
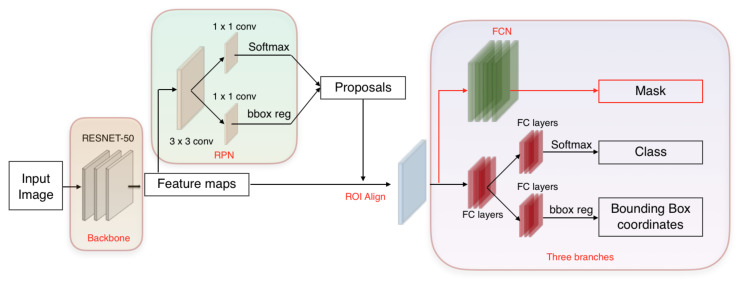
Overview of Mask RCNN architecture chosen to segment the pressure injury: Proposals about the regions which possibly contain the wound based on the input image are detected. Then three branches predict the class of the object, the bounding box and the mask in pixels of the wound. Both stages are connected to the backbone structure.

We propose an end-to-end framework which automatically retrieves quantitative information of the pressure injury using solely a 2D image and a 3D mesh of the wound, as shown in Figure 3. The developed system uses CNNs in order to automatically detect and segment the wound. Then it combines the segmentation results with the 3D mesh and automatically computes the wound depth, area, volumes and major and minor axes. Firstly, the 2D image is fed to a Mask-RCNN model in order to segment the pressure injury, as shown in Figure 4. Simultaneously, the 3D mesh is rasterized and a top view image and the matrix of face indices corresponding the top view are generated. After matching the top view image with the 2D image of the wound, captured using a camera, a projective transform matrix is calculated. Using the segmentation result, the top view image is segmented too, and the faces belonging to the inside and the boundaries of the wound are detected. The measurement block finally computes the depth, area, volume and axes of the wound.

### 3.2. Mask RCNN

The paper uses Mask RCNN algorithm [36] to detect and segment pressure injuries. It has very important research value and has broad application scenarios in the field of medical imaging. Mask RCNN is an instance segmentation framework which is an extension of Faster RCNN [37]. It is divided into two stages: the first stage is a small neural network called a Region Proposal Network (RPN) which scans the image and generates the proposals: the regions that most likely contain the wound. In the second stage, features are extracted from each proposed region in order to perform in parallel: proposal classification, bounding box regression and a binary pixel-level mask generation. The network structure block diagram of the Mask RCNN algorithm is shown in Figure 4.

### 3.3. Mesh Rasterization

**Figure 5:**
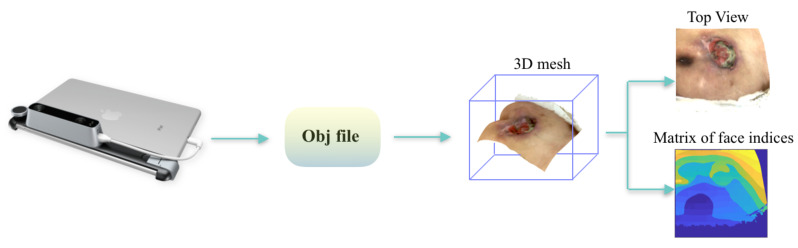
Mesh rasterization process: the Obj file of the 3D mesh acquired with the Structure Sensor is rendered in order to retrieve the top view image and the matrix of face indices.

When rendering the 3D mesh, we retrieve the top view image, where the surface of the pressure injury lies perpendicular to the camera direction, as shown in Figure 5. This step requires multiple transformations from world space to camera space, then to screen space. Firstly, the objects have point coordinates which are defined in world space, with respect to a global or world Cartesian coordinate system. The camera is no different than any other 3D object. By considering the camera as a reference system, points are transformed from the world coordinate system to the camera coordinate system, by multiplying the point world coordinates by the inverse of the local-to-world matrix. Then, to transform vertex coordinates from camera to screen-space, a so-called projection matrix is defined, which transforms coordinates to normalized device coordinates. All the aforementioned transformations are integrated within Pytorch3D library [38].

Then, we created a Phong renderer by composing a rasterizer and a shader. The textured Phong shader [39] interpolates the texture coordinates for each vertex from the texture image and applies the Phong lighting model.

Since the 3D meshes of the pressure injuries were acquired under the same conditions (near plane of the camera was always parallel to the surface of the wound, and the distance from the wound ranged between 30 to 40 cm), the selected camera viewpoint to retrieve the top view image was set to: Azimuth = 0, Elevation = 0 and Distance = 0.3 m. The corresponding tensor with the list of face indices which overlap with the pixels of the top view, was also generated. The shape of this tensor is (*image size, image size, faces per pixel*), where *faces per pixel* corresponds to the faces of the mesh which were projected into the same pixel in the output top view image. In our application, we chose *faces per pixel =* 5 in order to extract all the faces inside the mesh which are not seen in the top view image. This part of the framework was implemented using *PyTorch3D* library, an open-source toolkit for 3D based deep learning research [38].

### 3.4. Matching Block

Once the top view of the wound was generated, the next step was to match it with the 2D photograph of the wound. We used Progressive Sparse Spatial Consensus (PSSC) [40] as it has proven to be efficient in case the two images contain a large number of outliers.

In our application, the 2D image may contain several objects which are not present during the acquisition of the 3D mesh, such as: ruler, doctor’s hand, background, etc. In PSSC, the maximum likelihood spatial consensus estimation is done to formulate the matching, and the Expectation-Maximization (EM) approach is adopted for optimization.

**Figure 6:**
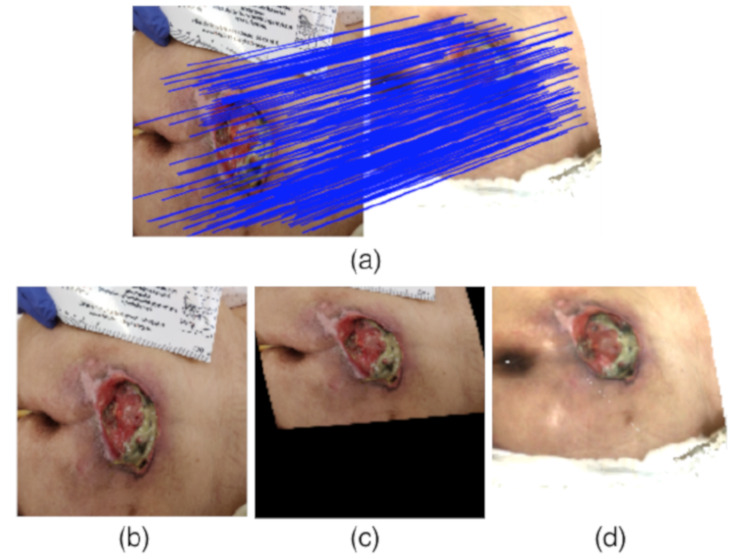
Matching illustration. (**a**) Point correspondence between 2D image and top view image, (**b**) 2D image, (**c**) 2D image after projective transformation to be aligned with the top view, (**d**) top view image.

Once the matching pixels from both images were retrieved, a projective transform matrix was calculated in order to transform the pressure injury mask from the 2D image to the top view. Using the pairs of matching landmarks in both images, the parameters of the matrix were calculated using the MATLAB function *fitgeotrans*, by specifying the desired transformation, which is projective in this case. This way, we could segment the pressure injury on the top view image by extracting the faces belonging to the boundaries and the inside of the wound, and proceed with the measurement block, as shown in Figure 6.

### 3.5. Measurement Block

**Figure 7:**
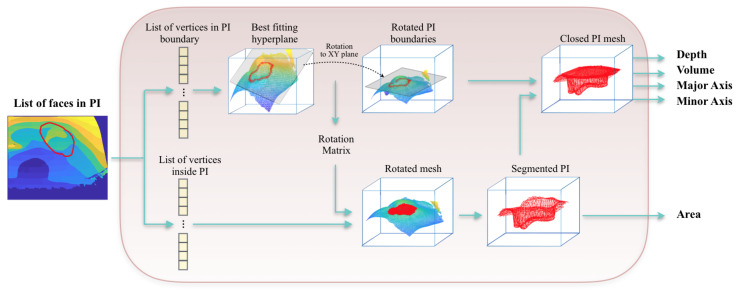
Schematic view of the measurement block: after extracting the vertices belonging to the boundaries and the inside of the wound, the 3D mesh is rotated and the best fitting hyperplane is calculated to close the surface of the pressure injury.

Using the matrix of face indices retrieved during the mesh rasterization, both the indices corresponding to the faces in the boundary and in the inside of the wound were extracted. For an easier processing of the mesh, we decided to rotate it in such way that the surface of the pressure injury is parallel to the XY axis, as shown in Figure 7. The best fitting hyperplane is calculated using RANSAC algorithm [41]. This algorithm tries to find the model that fits the maximum number of data points. Rather than using all the data once to obtain the solution and then eliminating the invalid data points, the RANSAC algorithm uses a small initial number of data points, estimates the model which fits the maximum number of inliers, then enlarges this set with consistent data and redo the estimations again until reaching the solution. Using the points in the 3D space which belong to the boundaries of the pressure injury, the RANSAC algorithm finds the maximum number of inliers which belong to the same hyperplane, which will be then the surface closing the top of the wound. Hence, the depth of each vertex inside the wound would be equal to its z-coordinate value—the z value of the hyperplane containing the boundary of the ulcer. The maximum absolute value of the resulting depths would represent the depth of the wound. The area of the wound is calculated by the summation of the areas of all the triangles inside the wound. In order to measure the volume, the mesh has to be closed. To achieve this, we created a new surface by computing the convex hull [42] on the xy coordinates of the vertices in the boundary of the wound. The height and width of the minimum bounding box enclosing this surface would represent minor and major axes. Then, the volume is measured by summing up the volumes of the tetrahedrons that go from the origin vertex, which was set to be the center of the closed top surface, to the triangles inside the mesh.

## 4. Experiments and Results

### 4.1. Data Collection

The database is composed of 210 photographs of pressure injuries. Then, 110 images were acquired from several hospitals in the region of Bilbao, Spain (Cruces Hospital, Santa Marina Hospital, Basurto Hospital), and 100 images from Medetec Medical Images online database (MIOD) [43]. All subjects gave their informed consent for inclusion before they participated in the study. The study was conducted in accordance with the Declaration of Helsinki, and the protocol was approved by the Ethics Committee of ETK-18/16-17.

**Figure 8:**
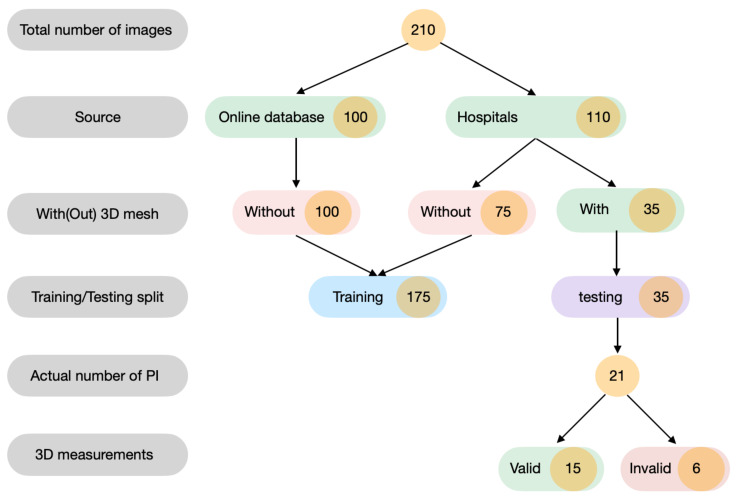
An illustrative diagram of the composition of the database used to conduct this research.

For the segmentation pipeline, 175 images were used for the training and 35 images were used for testing (the 35 images for testing contain 21 pressure injuries, as some PIs have several images), as shown in Figure 8. Those 21 pressure injuries were scanned using the Structure Sensor [11]. From those meshes, 6 meshes were not valid for the measurement of the depth and volume as they were either on the heel or on the hip, where the femur bone was pushing the inside of the ulcer above its surface, resulting in 15 meshes used for the 3D measurement. The 2D photographs which were captured during the hospital visits were acquired using a cell-phone camera (Oneplus 5T/ iPhone 6), with a resolution ranging from 1080 × 2280 to 2747 × 3079. No flash light was used, and the patients were moved in such way that the sunlight would reach the wound.

### 4.2. Implementation

We implemented the segmentation pipeline using Python 3.6 with *Keras 2.0* [44] and *TensorFlow 1.3* [45]. The backbones of Mask RCNN that were tried were ResNet50 and ResNet101. In addition, we tested the wound detection and segmentation capabilities of the model when trained on MS COCO and ImageNet datasets. The best performing backbone was ResNet-50, trained on MS COCO dataset. The best performing backbone was ResNet-50 and the number of epochs was set to 35. The 3D mesh processing and rasterization were done using *PyTorch3D* library and both the matching and measurement blocks were implemented using *Matlab 2018b*. We used a standard PC with dual Nvidia Geforce GTX 1080 Ti graphical processing unit (GPU) support.

### 4.3. Evaluation Metrics and Results

The segmentation method was evaluated using the following performance measures: Dice Similarity Coefficient (DSC), sensitivity and precision [46]. As for the measurement evaluation, we evaluated our results using the Mean Absolute Error (MAE) and Root Mean Squared Error (RMSE). The ground-truth measurements of the pressure injuries were made manually using Blender software. The mesh segmentation corresponded to the ground-truth segmentation of the 2D images, which was validated by two physicians who were told to independently examine the ground-truth images and document their professional criteria regarding our manual segmentation. First, faces, vertices and edges were selected to crop the pressure injury. Then, using the *Ruler* tool the minor and major axes were measured. Next, from the *3D printing tab*, the *Area* tool from *Statistics* was used to measure the area of the wound. Subsequently, in order to close the mesh, the *Remesh* tool was used, and the number of octrees was set to 6 in order to maintain the exact shape of the wound with a smooth closed surface. Similarly to area and axes measurement, the values of the volume and depth were retrieved using *Volume* tool and *Ruler* tool, respectively.

**Figure 9:**
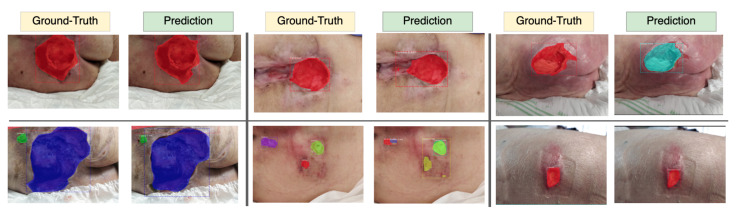
Results of the segmentation of the pressure injuries (Colors were generated randomly).

The best results of segmentation were achieved using Mask-RCNN model with ResNet-50 backbone, with a mean Dice score of 0.83 for wound segmentation, a mean sensitivity score of 0.85 and a mean precision score of 0.87. Figure 9 depicts some segmentation results.

**Figure 10:**
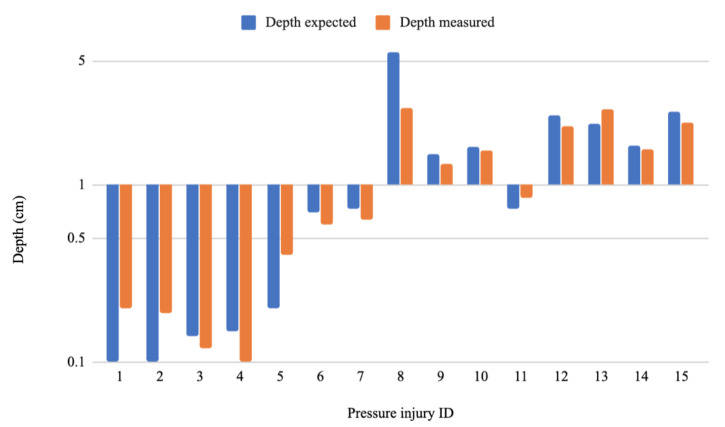
Measurement results of 15 different pressure injuries: depth in logarithmic scale (cm).

**Figure 11:**
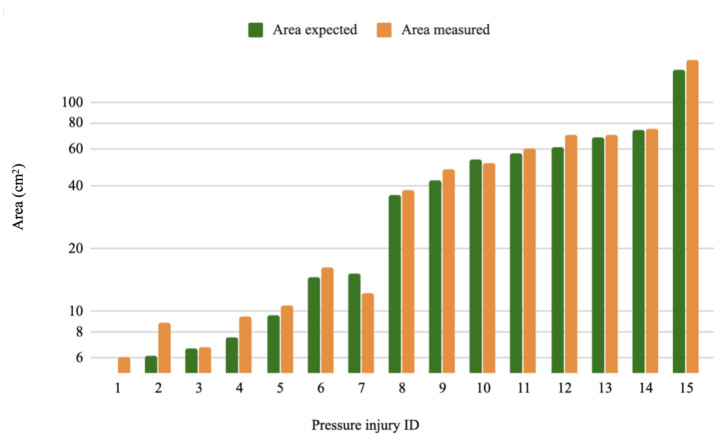
Measurement results of 15 different pressure injuries: area in logarithmic scale (cm^2^).

**Figure 12:**
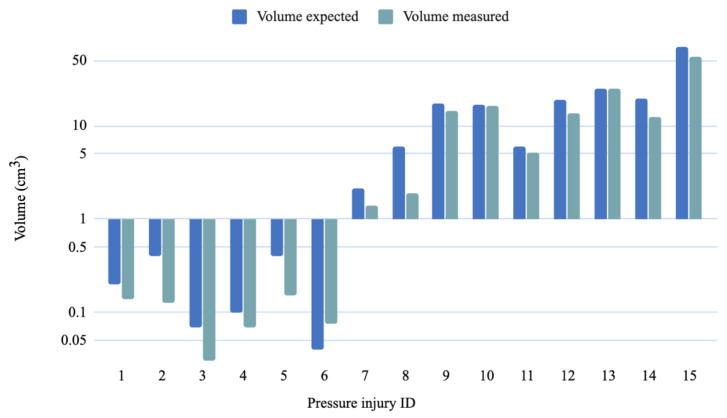
Measurement results of 15 different pressure injuries: volume in logarithmic scale (cm^3^).

**Figure 13:**
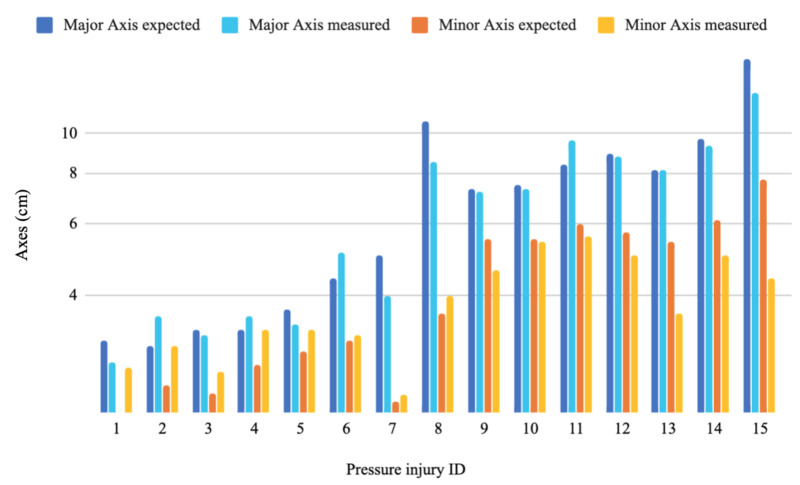
Measurement results of 15 different pressure injuries: minor and major axes in logarithmic scale (cm).

As for the measurements part, the MAE values were 0.74 cm, 4.69 cm^3^, 5.41 cm^2^, 1.03 cm and 1.10 cm for the depth, volume, area, major axis and minor axis respectively. Whereas the RMSE values were 0.58 cm, 1.57 cm^3^, 1.86 cm^2^, 0.82 cm and 0.87 cm for the depth, volume, area, major axis and minor axis, respectively. Figure 10, Figure 11, Figure 12 and Figure 13 show four bar plots corresponding to the measurements of the depth, volume, area, major axis and minor axis, respectively, on the 15 pressure injuries used for validation. Since the pressure injuries had different sizes and shapes, the plots were chosen to have a logarithmic scale, in order to have a better view of those with a value less than 1 (cm/cm^2^/cm^3^). The pressure injury IDs are consistent in all the plots, where we chose to order them by their real areas.

**Figure 14:**
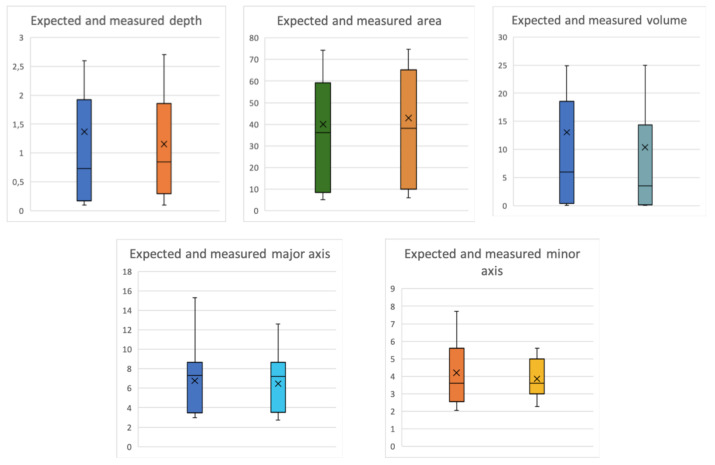
Boxplots of the five components (expected vs. measured).

Figure 14 shows the box plots of the five components measured. Each subplot contains the distribution patterns of both the expected and measured components. We can notice from all the plots that the distribution of the measured values are similar to the expected ones. However, for both major and minor axes, maximum non-outlier values are shifted by almost 2 cm, which is due to some irregular borders of some pressure injuries that tend to cause also uncertainty in the segmentation task.

## 5. Discussion and Conclusions

In this paper, we presented an effective framework for an automatic segmentation and measurement of pressure injuries. By automatically combing the information from high resolution photograph and 3D meshes, we were able to achieve very close results to those retrieved manually using Blender, which requires time and software manipulation expertise. Unfortunately, healthcare professionals and caregivers generally lack these two requirements. Hence, this system will enable them to automatically and non-intrusively retrieve the measurements of the pressure injuries and eventually track their healing evolution.

For the segmentation part, the state-of-the-art contributions were largely achieved using classical image processing and machine learning techniques. Since the databases used differ from one to another, as well as the number of images used, a comparison between the techniques could be misleading. Moreover, many researchers present their segmentation results using the accuracy. This metric is highly misleading as a high value of pixel accuracy does not always imply superior segmentation ability. The following contributions in the literature have stated their recall and Dice coefficient scores, which are more reliable metrics. Dhane et al. [47] used 105 Lower extremity wound images, preprocessed them using color correction and denoising and applied spectral clustering for wound segmentation, and applied morphological post-processing. The spectral clustering showed an average sensitivity 89.54%. Veredas et al. [48] used 113 images of pressure injuries for training and 322 for testing, applied statistical color models from a Bayesian perspective to estimate the probability of a pixel to belong to the ROI and achieved an F-score of 73.89%. Wannous et al. [49] used 50 images of all types of wounds and reached an average overlap score of 73.1%.

**Figure 15:**
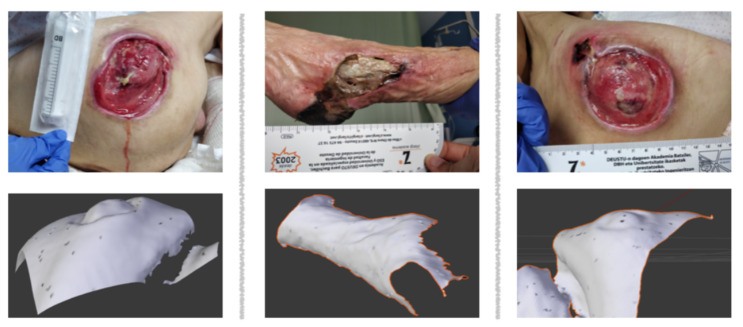
Example of pressure injuries invalid for the measurement of the volume: Left and right columns present two pressure injuries located on the hip bone, whereas the middle column present a pressure injury located on the heel and ankle.

It is to be noted that the measurement of the volume in our system is relevant when the pressure injury is on a concave surface which can be closed without altering the shape of the wound. For instance, on Figure 15, both pressure injuries on the left and right are on the hip bone of the patient. The femur bone is above the surface, which creates a concave surface inside the wound. The pressure injury in the middle is located on the heel and ankle. As this surface is concave too, the center of the pressure injury will be located above the surface containing the boundaries of the wound. Hence, summing all tetrahedra bounded by the origin and a mesh triangle will result in adding more subvolumes inside the concave surface, which do not originally belong to the wound. Consequently, the result will be erroneous.

Despite the fact that the segmentation precision was solely 87%, which is mainly due to the ambiguous borders of some pressure injuries, the matching block slightly enlarges the borders of the mask in order to ensure that the whole wound is considered for the measurement. The ruler can also be detected in case the 3D mesh is not provided. This way, the area and diameters of the wound could be measured too. This part will be added to our proposed framework, that is currently being integrated in an interactive system with a user-friendly interface, which will be used during the future hospital study visits. This way, we will enlarge the database and reinforce the learning of our system.

Most hospitals require documentation of the pressure injury using images, and additionally, expect the healthcare professionals to take more detailed information. Using this system, they will have the ability to use the time with the patient more effectively. In order to independently use this system, medical centers will need to acquire the Structure Sensor, along with the mobile device to mount it on. The total acquisition time could range from 1 to 2 min, for both the 2D image and 3D mesh combined. Our experience with caregivers during study visits demonstrated that they can capture the 3D meshes efficiently. The data captured will then be processed using the user-interface integrated within the device. Some training for the acquisition of the 3D meshes will be given. Hence, we will provide both a document and a video explaining the process.

## Figures and Tables

**Table 1:** Contributions in pressure injury segmentation and measurement.

	[31]	[23]	[32]	[33]	[34]	[35]	[28]	[29]	[30]	Ours
**Wound segmentation**	✔	✔	✔	✔	✔	✔	✔	✔	✔	✔
**Pixel-wise segmentation**	✔	✔	✔			✔	✔	✔	✔	✔
**Area in real world units**							✔	✔	✔	✔
**Volume in real world units**										✔
**Depth in real world units**										✔

## Abbreviations

The following abbreviations are used in this manuscript:

SLS	Structured Light System
SOC	System On a Chip
IR	Infrared
Mask RCNN	Mask Regional Convolutional Neural Network
PI	Pressure Injury
PSSC	Progressive Sparse Spatial Consensus
EM	Expectation-Maximization
RANSAC	RANdom SAmple Consensus
MIOD	Medetec Medical Images Online Database
SIFT	Scale Invariant Feature Transform
SVM	Support Vector Machines
FCN	Fully Convolution Network
RPN	Region Proposal Network
FC layers	Fully Connected layers
ROI	Region Of Interest
bbox	Bounding Box
